# An unusual case of Behcet disease with posterior scleritis: A case report

**DOI:** 10.1097/MD.0000000000016886

**Published:** 2019-08-30

**Authors:** Chihiro Yanagida, Yoshihiko Usui, Jun-Ichi Sakai, Hiroshi Goto

**Affiliations:** Department of Ophthalmology, Tokyo Medical University, Tokyo, Japan.

**Keywords:** anti-tumor necrosis factor-alpha (anti-TNF-α) therapy, Behcet disease, case report, posterior scleritis

## Abstract

**Rationale:**

Posterior scleritis is an ocular inflammatory disorder that can be associated with both infectious and non-infectious immune reactions. Behcet disease is a chronic, relapsing, multisystemic inflammatory disorder with uveitis. There are no reported cases of posterior scleritis with Bechet disease.

**Patient concerns:**

A 50-year-old man previously diagnosed with systemic Behcet disease presented with ocular pain and decreased vision in the left eye.

**Diagnosis:**

Posterior scleritis associated with Behcet disease was diagnosed based on optical coherence tomography showing choroidal folds, as well as contrast computed tomography and ultrasound sonography demonstrating thickening of the posterior sclera.

**Interventions:**

Treatment with systemic corticosteroids was initiated. Since inflammation relapsed during steroid tapering, anti-tumor necrosis factor-alpha (TNF-α) therapy was used in combination, and tapering of steroids was possible without recurrence of inflammation for 12 months.

**Outcomes:**

Posterior scleritis was resolved and visual acuity improved. With the continuation of TNF-α therapy, oral prednisolone was successfully tapered and discontinued. No relapse of inflammation was observed at follow-up 1 year after discontinuation of prednisolone.

**Lessons:**

Ophthalmologists should be aware of the possibility of rare manifestation of posterior scleritis in patients with Behcet disease, and that combined use of systemic steroids and anti-TNF-α therapy may resolve the scleritis without recurrence of inflammation.

## 1 Introduction

Posterior scleritis is an ocular inflammatory disorder that is predominantly idiopathic, autoimmune, or rarely, infective.^[1]^ On the other hand, Behcet disease is a chronic autoimmune disease with unknown etiology, which causes inflammation in multiple organs including skin, mucous membranes, and nerves, resulting in functional and organic disorders.^[2]^ The most common clinical signs of ocular manifestations of Bechet disease are hypopyon, macular edema, and retinal vasculitis.^[3]^ In general, scleritis may complicate rheumatoid arthritis and ANCA-associated vasculitis, but the association of scleritis with Behcet disease is rare.^[4]^

To the best our knowledge, there are no published reports of posterior scleritis associated with Behcet disease to date. In this report, we describe a case of posterior scleritis in a patient diagnosed with systemic Behcet disease.

## 2 Case presentation

A 50-year-old man presented with left ocular pain and decreased vision. He was diagnosed with Behcet disease positive for HLA A-26 in 2006, and had been treated in our hospital with colchicine and topical corticosteroid for inflammatory attacks.

**Figure 1:**
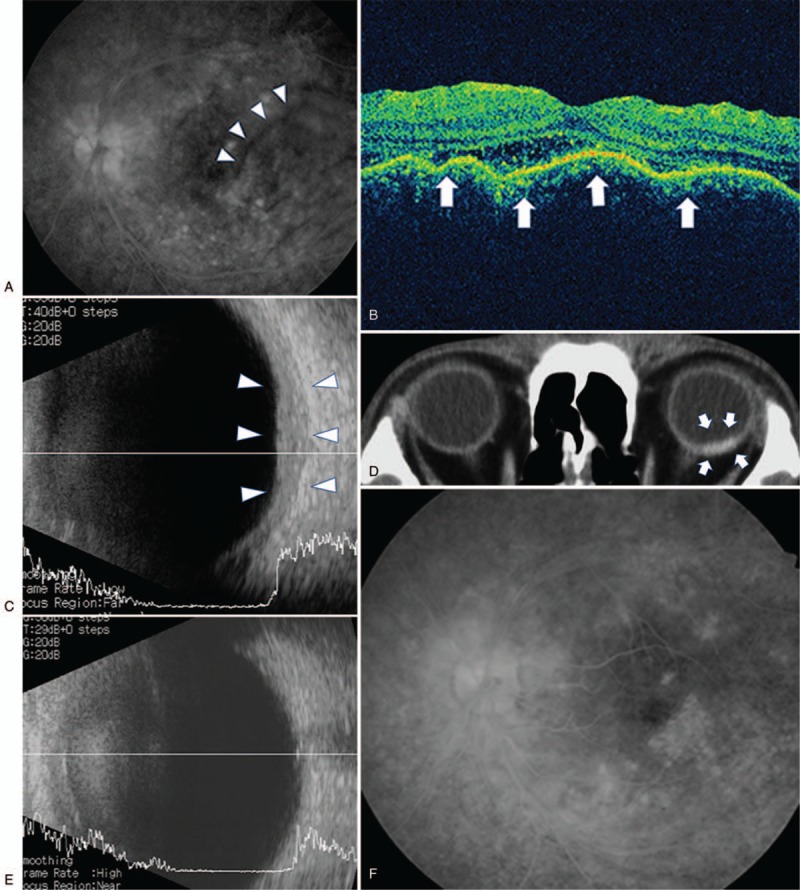
(A) Fluorescein angiography of the left eye showing peripheral vasculitis characteristic of Behcet disease and retinal folds at the posterior pole. (B) Optical coherence tomography showing choroidal folds (arrows) in the left eye. (C) B-scan ultrasonography of the left eye demonstrating thickened sclera (arrowheads). (D) CT scan of the patient showing contrast enhancement (arrows) localized to the posterior sclera of the left eye. (E) B-scan ultrasonography of the left eye 43 weeks after treatment showing disappearance of choroid hypertrophy. (F) Fluorescein angiography of the left eye 43 weeks after treatment showing disappearance of retinal folds.

At presentation, his corrected visual acuity was 20/25 in the right eye and 20/200 in the left eye, and intraocular pressure was normal. The anterior chamber and vitreous body of the left eye showed 1+ inflammatory cells and fundus examination revealed redness of the optic disc in both eyes and focal choroidal folds in the left eye. Fluorescein angiography revealed leakage from the optic disc in both eyes and mild linear hypo-fluorescence at the posterior pole of the left eye (Fig. 1A). Optical coherence tomography showed a serous macular detachment in the right eye and choroidal folds in the left eye (Fig. 1B). Ultrasonography showed thickening of the posterior sclera (Fig. 1C). Orbital contrast computed tomography showed a contrast-enhanced focus localized to the posterior sclera (Fig. 1D). In hematological and biochemical examinations, there were no increases in antinuclear antibody, antineutrophil cytoplasmic antibody, P-ANCA, C-ANCA, rheumatoid factor, HLA B-27, inflammatory response, and angiotensin-converting enzyme, but an increase in complement (CH50 55 U/ml) was observed. Serological tests for infectious diseases (herpes simplex, zoster, syphilis, and tuberculosis) were negative. Based on these findings, this case was diagnosed as posterior scleritis associated with Behcet disease.

Systemic steroid therapy was initiated for the treatment of posterior scleritis, while treatment for Behcet disease was not changed. Oral prednisolone (0.4 mg/kg) was prescribed and visual acuity improved gradually. However, when prednisolone was tapered to a dose of 0.2 mg/kg, the patient complained of blurred vision in the left eye caused by recurrence of inflammation probably due to Behcet disease. Therefore, anti-TNF-α therapy using adalimumab (40 mg every 2 weeks) was added to prednisolone. Consequently, with the continuation of adalimumab, prednisolone was successfully tapered and discontinued without relapse of inflammation. The treatment duration of oral prednisolone was 12 months and that of adalimumab was 21 months; the total duration of treatment was 21 months. At 12 months after oral prednisolone treatment cessation (2 years after onset), no recurrence was observed and visual acuity was maintained at 20/20. Ultrasonography revealed the resolution of thickening of the posterior sclera (Fig. 1E). The disappearance of linear fluorescence was confirmed on fluorescein angiography (Fig. 1F).

## 3 Discussion

Posterior scleritis was reported to be associated with systemic diseases in 38% of cases by Lavric et al,^[1]^ in 29% by McCluskey et al,^[5]^ and in 19% by Gonzalez et al.^[6]^ Review of the literature revealed that posterior scleritis is most frequently accompanied by rheumatoid arthritis.^[1,5]^ In the present case, although Behcet disease was diagnosed in the past, blood tests at presentation showed no evidence of systemic diseases that could cause posterior scleritis. Although the mechanism by which the inflammatory process in Behcet disease induces scleritis remains unclear, necrotizing scleritis and nodular scleritis secondary to Behcet disease have been reported.^[7,8]^ Development or aggravation of scleritis has been reported to be associated with generalized vasculitis disease.^[9]^ As vasculitis is the underlying pathology of Behcet disease, there is a possibility that scleritis develops as a complication of Behcet disease.^[8]^ We speculate that the same mechanism may apply to posterior scleritis associated with Behcet disease as seen in the present case. However, there are no reports of posterior scleritis associated with Behcet disease. Therefore, diagnosis of posterior scleritis, in this case, was challenging, but the diagnosis was possible by ultrasonography, fluorescein angiography, and optical coherence tomography.^[10]^ Orbital contrast computed tomography has been reported to be useful for the diagnosis of posterior scleritis,^[11]^ and also contributed to the diagnosis in this case.

Systemic corticosteroid therapy is the mainstay of management for posterior scleritis. There is no clear consensus on specific corticosteroid dosing regimen for this condition. Moreover, systemic corticosteroid therapy is still under debate because of the risk of exacerbation of Bechet disease during the planned tapering of systemic corticosteroid therapy.^[12]^ In our case, inflammation relapsed during conventional oral steroid treatment for posterior scleritis, suggesting a high possibility that posterior scleritis developed with Behcet disease as the underlying cause. Therefore, the combined use of TNFα therapy was necessary during tapering of systemic steroid.

Many cytokines are activated in the presence of chronic inflammation. Pharmacological blockade of certain cytokines is an important approach in the control of inflammation. TNF, one of the inflammatory cytokines, has been shown to control chronic inflammation,^[13–15]^ and has also be reported to be involved in the pathogenesis of Behcet disease.^[16–18]^ Therefore, anti-TNFα therapy is considered useful for the treatment of Behcet disease.

The safety and efficacy of anti-TNF therapy for the management of ocular manifestations (including scleritis) of inflammatory diseases including Behcet disease have been reported.^[19,20,21]^ In the present case, after initial treatment with high dose systemic corticosteroids, tapering resulted in a relapse of intraocular inflammation, and combined treatment with anti-TNF-α therapy allowed safe and successful tapering prednisolone and improved visual acuity. To date, the standard treatment for scleritis with Behcet disease has not been established, probably due to the rarity of the disease. Anti-TNF therapy seems to be a safe and effective option for the treatment of scleritis associated with Behcet disease.

In conclusion, we have reported a rare case of posterior scleritis associated with Behcet disease. This case indicates that ophthalmologists should be aware of the possibility of rare manifestation of posterior scleritis in patients with Behcet disease.

## Author contributions

**Conceptualization:** Yoshihiko Usui.

**Data curation:** Chihiro Yanagida, Yoshihiko Usui.

**Formal analysis:** Yoshihiko Usui.

**Funding acquisition:** Yoshihiko Usui.

**Investigation:** Yoshihiko Usui.

**Methodology:** Yoshihiko Usui.

**Project administration:** Yoshihiko Usui.

**Resources:** Yoshihiko Usui.

**Software:** Chihiro Yanagida, Yoshihiko Usui.

**Supervision:** Yoshihiko Usui, Jun-ichi Sakai, Hiroshi Goto.

**Validation:** Hiroshi Goto.

**Visualization:** Chihiro Yanagida, Yoshihiko Usui.

**Writing – original draft:** Chihiro Yanagida, Yoshihiko Usui.

**Writing – review & editing:** Chihiro Yanagida, Yoshihiko Usui.
